# Chemical bonding in aqueous hexacyano cobaltate from photon- and electron-detection perspectives

**DOI:** 10.1038/srep40811

**Published:** 2017-01-18

**Authors:** Sreeju Sreekantan Nair Lalithambika, Kaan Atak, Robert Seidel, Antje Neubauer, Tim Brandenburg, Jie Xiao, Bernd Winter, Emad F. Aziz

**Affiliations:** 1 Institute of Methods for Material Development, Helmholtz-Zentrum Berlin für Materialien und Energie, Albert-Einstein-Strasse 15, 12489, Berlin, Germany; 2 Freie Universität Berlin, Fachbereich Physik, Arnimallee 14, D-14195, Berlin, Germany; 3 School of Chemistry, Monash University, Clayton, 3800, VIC, Australia

## Abstract

The electronic structure of the [Co(CN)_6_]^3−^ complex dissolved in water is studied using X-ray spectroscopy techniques. By combining electron and photon detection methods from the solutions ionized or excited by soft X-rays we experimentally identify chemical bonding between the metal center and the CN ligand. Non-resonant photoelectron spectroscopy provides solute electron binding energies, and nitrogen 1 s and cobalt 2p resonant core-level photoelectron spectroscopy identifies overlap between metal and ligand orbitals. By probing resonances we are able to qualitatively determine the ligand versus metal character of the respective occupied and non-occupied orbitals, purely by experiment. For the same excitations we also detect the emitted X-rays, yielding the complementary resonant inelastic X-ray scattering spectra. For a quantitative interpretation of the spectra, we perform theoretical electronic-structure calculations. The latter provide both orbital energies and orbital character which are found to be in good agreement with experimental energies and with experimentally inferred orbital mixing. We also report calculated X-ray absorption spectra, which in conjunction with our orbital-structure analysis, enables us to quantify various bonding interactions with a particular focus on the water-solvent – ligand interaction and the strength of π-backbonding between metal and ligand.

Electronic structure of transition-metal (TM) complexes has been an area of active research for nearly a century^1^,^2^. More recent studies, motivated by the global demand for clean and sustainable energy, have spurred the field of energy material research, promoting the identification and synthesis of various TM complexes for applications in solar energy harvesting and various catalytic processes. At the heart of these processes are the ultrafast charge-transfer processes leading to the formation and breaking of chemical bonds. In the case of TM complexes bond formation mainly involves the sharing of electron densities between valence 3d orbitals of the metal atom and the outer ligand orbitals. In TM–cyanide (CN^−^) complexes, which are studied here, two types of covalent bonds are operative, the σ- and π-bonds. Such interactions are common in organometallic chemistry involving TM ions with multi-atomic ligands. In σ-bonding (also called σ-donation), the electron density from the valence CN^−^ orbitals is transferred to the unoccupied metal orbitals. The π-bonding arises due to the transfer of electron density from the occupied metal orbital to the unoccupied CN^−^ orbitals, commonly known as the π-backbonding. It is a result of the strong π-acceptor nature of the CN^−^ ligands, relieving the metal of excess negative charge, and leading to the stabilization of the complex. The chemical properties of TM complexes strongly depend on the type and extent of bonding, and hence on electronic structure. Therefore, the fundamental understanding of different types of bonding interactions is important in tailoring and tuning material properties for given applications such as catalytic water splitting^3^. With the advent of liquid-microjet^4^ and flow-cell^5^ methods, combined with state-of-the-art synchrotron based X-ray spectroscopic techniques, it is now possible to explore the electronic structure in aqueous environments^6^. This is even crucial in the case of multi-atomic ligands when the metal center is not in direct contact with the solvent.

In the present work we explore the local electronic structure and bonding interactions of the [Co(CN)_6_]^3−^ complex in aqueous solutions using X-ray spectroscopic methods detecting both emitted photons and electrons. [Co(CN)_6_]^3−^(aq), with low-spin 3d^6^ electronic ground-state configuration in an octahedral strong ligand field^2^, is a particularly interesting case for exploring the strength of π-backbonding and σ-bonding because the t_2g_ and e_g_ valence levels resulting from the splitting of the 3d levels by the ligand field are completely filled and completely empty, respectively. σ bonding in [Co(CN)_6_]^3−^(aq) arises from the donation of electron density from the highest occupied molecular orbital (HOMO), 5σ, of CN^−^ to the metal 3d orbitals. π-bonding results from the filling of the vacant 2π* orbitals, i.e., the lowest unoccupied molecular orbitals (LUMO) of the CN^−^ by metal 3d (t_2g_) electrons^7^,^8^. It is worth noting that the CN^−^ ligand is isoelectronic to CO, hence the ligand fields are of similar magnitude leading to comparable σ-donation and π-acceptor bonding^9^,^10^.

Experimentally, we measure the Co L_2,3_ (2p) and N K (1 s) edge partial-fluorescence yield (PFY) and partial-electron yield (PEY) X-ray absorption (XA) spectra, and in addition resonant inelastic X-ray scattering (RIXS), valence and core-level photoelectron spectra are reported. For an interpretation of the experimental PFY- and PEY-XA spectra we use density functional theory/restricted open-shell configuration interaction singles (DFT/ROCIS) for the L-edge and time-dependent density functional theory (TDDFT) calculations for the K-edge^11^. Several studies on the electronic structure of various TM cyanides, in aqueous and ethanol solutions, using UV/Vis spectroscopy^12^,^13^, have been previously reported. Also, metal L-edge and nitrogen K-edge total electron yield (TEY-XA) measurements have been performed from powder samples of TM–cyanide [X(CN)_6_]^3−^ complexes, X = Cr, Mn, Fe, and Co^14^. Other studies have explored the electronic structure of ferro- and ferricyanide complexes applying L-edge TEY-XA^15^ and K-edge RIXS^16^ spectroscopy, respectively, also using powders.

Regarding the photoelectron (PE) spectroscopy measurements performed here, we report resonant (RPE spectra) cobalt L-edge and nitrogen K-edge spectra, as well as non-resonant valence photoelectron spectra from [Co(CN)_6_]^3−^ aqueous solution. The RPE spectra provide unique insight into mixing of metal and ligand orbitals, and furthermore integration of the signal intensities of RPE spectra measured across a given resonance yields the respective XA spectra. Here one essentially tracks the intensity of the Auger electrons emitted in a given autoionization channel (valence to core hole) which is to a first approximation proportional to the actual X-ray absorption^17^. An important aspect in the present work is the exploration of signal enhancements at resonant excitation (due to interfering Auger electrons and direct photoelectrons with the same kinetic energies) which identifies metal and ligand orbital overlap^18^. RIXS measurements are performed at the same edges, Co 2p and N 1 s. RIXS and XAS, being element/site specific photon in –photon out techniques, exploit core level excitations to probe occupied and unoccupied electronic states of the investigated molecule, respectively^19^,^20^. Analogous to the RPE spectra, RIXS spectra shed light into metal-ligand orbital hybridization and integration of the RIXS spectra yields the PFY-XA spectra. Analysis of all mentioned electron- and photon-out channels is crucial for an accurate understanding of the complex electronic-structure interactions of the [Co(CN)_6_]^3−^(aq) complex.

## Methods

### Experiment

Potassium hexa-cyano cobaltate(III) was purchased from Sigma Aldrich (purity > 97%). A 200 mM aqueous solution, using Milli-Q water, was freshly prepared immediately prior to experiments. The partial-fluorescence yield (PFY) XA, RIXS and (non-resonant) X-ray emission (XE) spectroscopy measurements at the Co L_2, 3_-edge and N K-edge were carried out with the LiXEdrom^21^ setup, and partial-electron yield (PEY) XA spectroscopy and (resonant) photoelectron spectroscopy (RPES) measurements were carried out with the LiquidPES^4^,^22^ setup at the U41-PGM undulator beamline at the BESSY II synchrotron facility, Berlin. The photon-out detection the measurements were performed using a flow-cell^5^, whereas for the electron emission a liquid microjet^22^ was employed. Both flow-cell and microjet allow for renewal of sample during exposure to synchrotron radiation which minimizes sample damage. The liquid jet (24 μm diameter) was injected into the vacuum chamber from a fused-silica nozzle. The jet velocity and the sample reservoir temperature were approximately 40 ms^−1^ and 12 °C, respectively. The actual jet temperature in the interaction region is lower than 12 °C but an exact value cannot be provided; except the lower limit of 3 °C, as estimated from evaporative cooling modeling^23^,^24^. Photoelectrons were detected at the magic angle, i.e., the angle between the synchrotron-light polarization axis and the electron analyzer was set to 54.7°. Electrons emitted from the liquid jet enter the differentially-pumped electron analyzer through a 500 μm orifice. Under operation conditions the vacuum in the liquid-jet main chamber is 10^−4^ mbar, and 10^−7^ mbar in the hemispherical electron energy analyzer equipped with a multichannel detector. A small distance of <0.5 mm between the liquid jet and the orifice of the spectrometer assures that detected electrons have not suffered from inelastic scattering with gas-phase water molecules near the jet surface which enables the aqueous-phase photoemission studies^23^. The energy resolution of the U41-PGM beamline was better than 40 meV for 200 eV photon energies used for the valence PE measurements, better than 240 meV for the N 1s absorption measurements near 400 eV, and 600 meV for the Co L-edge absorption measurements at photon energies above 780 eV; the same beamline resolution applies also for the RIXS measurements. The energy resolution of the hemispherical analyzer was constant with kinetic energy, ~100 meV at 10 eV pass energy (used for the valence PE spectroscopy measurements), and ~200 meV at 20 eV pass energy (used for all other (resonant) PE spectroscopy measurements).

The flow cell of the LiXEdrom setup was equipped with a SiC membrane of 150 nm thickness. Transmission of this window for the given experimental geometry is approximately 10% for 400 eV photons, and 65% for 800 eV photons. Pressure in the main chamber was in the mid 10^−6^ mbar range. The X-ray fluorescence spectrum has been recorded using a detector mounted perpendicular with respect to the direction of the incident X-rays. The emitted X-rays are diffracted using a grating (7.5 m radius, 1200 lines/mm), and the energy-dispersed light is detected using a MCP/fluorescence screen/CCD camera stack operated at 10^−8^ mbar. Sample, grating, and detector were arranged in Rowland-circle geometry.

## Computations. 

The calculations were carried out with the ORCA software package^25^. Molecular geometry optimizations were performed using the M06^26^ density functional method with the def2-TZVP(-f) basis set^27^. Transition energies and moments for the Co L-edge were calculated with DFT/ROCIS using the same basis set and B3LYP^28^,^29^ functional. For the N K-edge, TDDFT method was employed, again using the same functional and basis set. For DFT/ROCIS calculations, we used the B3LYP functional with the parameters c_1_ = 0.18, c_2_ = 0.20, and c_3_ = 0.40^11^. During the calculations, the resolution of identity^30^,^31^,^32^,^33^,^34^ approximation was used employing the def2-TZV/J auxiliary basis set^35^. Numerical integrations during the DFT calculations were performed on a dense grid (ORCA grid 5). L-edge and K-edge absorption spectra were obtained by applying a 0.8 eV and 0.4 eV Gaussian-type broadening on each transition moment respectively. The geometry calculations had no symmetry constraint. For XES calculations we used the B3LYP functional along with the def2-TZVP(−f) basis set and the integrations were carried out on a dense grid (ORCA grid 5). In all calculations, the relativistic effects were taken into account using zeroth order regular approximation (ZORA)^36^. The solvent effects were accounted for using the conductor-like screening model (COSMO water) in ORCA^37^.

## Results and Discussion

We start by presenting the valence photoelectron spectrum of aqueous K_3_[Co(CN)_6_] along with the neat-water spectrum. From the difference spectrum, solution minus water, the spectral contributions from the [Co(CN)_6_]^3−^ anion and the K^+^ cation can be identified. We then report the RPE spectra, although the initial focus is on the signal-integrated RPE spectra, i.e., the Co 2p and N 1s PEY-XA spectra. The discussion of the specific signal enhancements in the RPE spectra can then be built on the features of the XA spectra. An analysis of the nature of specific valence electronic states, based on ground-state DFT calculations, interpretation of Co L-edge and N K-edge RIXS assisted by XES calculations, and interpretation of Co L-edge and N K-edge XA spectra based on DFT/ROCIS and TDDFT methods, respectively, are presented thereafter.

## Experimental Results

### K_3_[Co(CN)_6_](aq) valence photoelectron spectra

**Figure 1:**
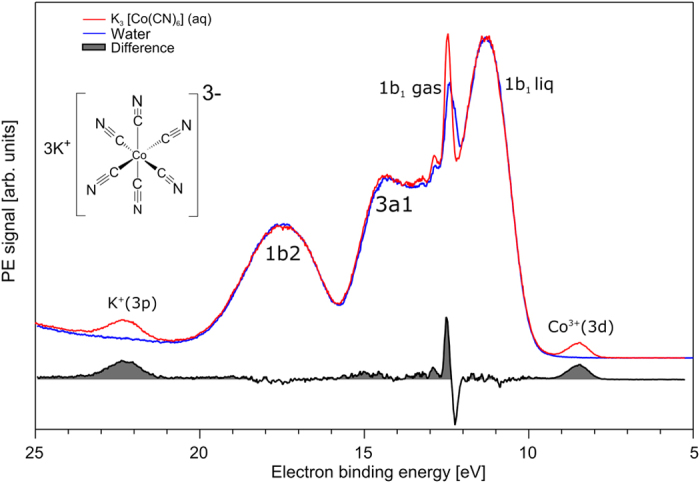
Valence photoelectron spectra of 200 mM K_3_[Co(CN)_6_] aqueous solution (red) and pure water (blue). Both spectra are measured at 200 eV photon energy. The lower tier shows the difference spectra of the former from the water spectrum. The structural formula of the complex is shown in inset.

Figure 1 shows the valence PE spectrum of a 200 mM K_3_[Co(CN)_6_] aqueous solution (in red) measured at a photon energy of 200 eV. For reference, we also show the spectrum of neat liquid water (in blue), obtained under identical conditions. Energies are presented as electron binding energies (BE). The peaks at 11.3, 14.35, 17.50 eV BE in the water spectrum are due to the ionization of the three water valence orbitals, 1b_1_, 3a_1_, and 1b_2_ 38. The strong narrow peak at 12.45 eV arises from the 1b_1_ orbital of gaseous water, and the small narrow peaks at 12.9 and 13.2 eV correspond to 1b_1_ vibrational states. The bottom tier is the difference spectrum, subtracting water from solution spectrum, which reveals several solute contributions. Most clearly visible is the signal due to the Co^3+^ 3d states, centered near 8.5 eV BE, and spread over a 1 eV range. Since the cobalt e_g_ state is empty, the 8.5 eV peak is entirely assigned to the fully occupied t_2g_ level, and represents ionization of the highest occupied molecular orbital (HOMO) of [Co(CN)_6_]^3−^(aq). This value is approximately 2.5 eV lower than the ionization of the highest occupied orbital of neat liquid water. The peak at 22.2 eV is due to ionization of the K^+^(3p) state^22^. We show below that solute-derived spectral contributions are more visible in the RPE spectra, which moreover reveal additional spectral features.

### Co L-edge valence RPE map and spectra

**Figure 2:**
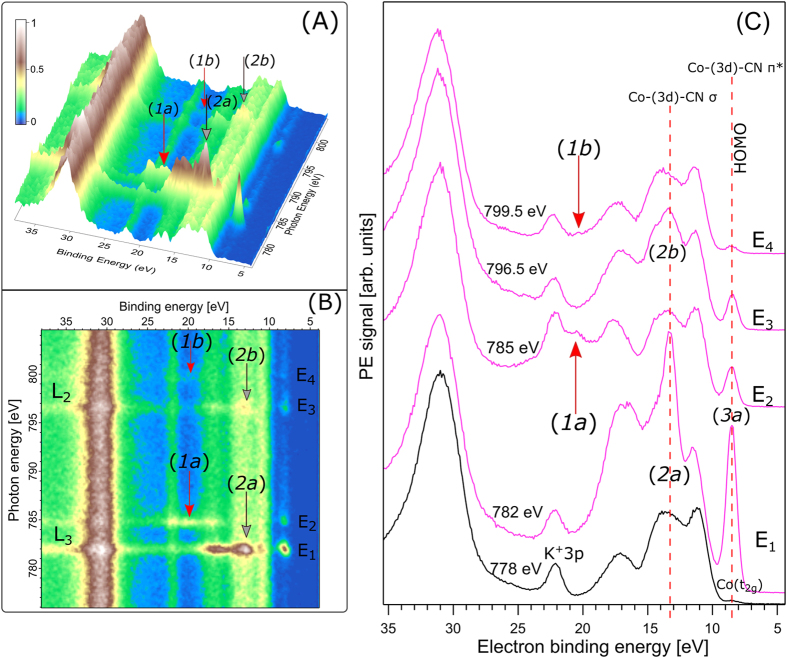
RPE spectra of 200 mM K_3_[Co(CN)_6_] aqueous solution obtained upon tuning the photon energy across Co L-edge. (**A**) Shows electron intensity in a 3D landscape on the binding energy scale; the important signal enhancements at resonance energies are marked in red (***1a, 1b***) and black (***2a, 2b***) arrows. (**B**) Presents the respective 2D plot. E_1_, E_2_, E_3_, and E_4_ mark the resonant excitations at L_3_ and L_2_ edges. (**C**) Illustrates the off-resonant (black) and all other resonant (pink) excitations. The vertical dotted lines and arrows mark the different resonant-enhanced states of interest.

Co L-edge (2p–3d excitation) RPE spectra from the same 200 mM solution as in Fig. 1 are shown in Fig. 2. The 3-dimensional landscape plot of Fig. 2A displays the electron-signal intensity as a function of both (resonant) photon energy and electron binding energy; with a 0.15 eV step width in excitation photon energy the map has been acquired within 35 minutes. Figure 2B presents the same data in two dimensions, but with the signal intensity expressed by color codes. Figure 2C shows several individual RPE spectra, each measured at the photon energy labeled E_1_–E_4_ in Fig. 2B, marking the most noticeable resonant signal enhancements. This is the case at 13.2 eV BE (labels ***2a, 2b***) for excitation photon energies 782 (E_1_)/796.5 eV (E_3_), and at 20.5 eV BE (labels ***1a, 1b***), although the feature is much weaker, for 785 (E_2_)/799.5 eV (E_4_) excitation, respectively. We will use the notations E_1_–E_4_ throughout the text when referring to the main maxima of cobalt X-ray absorption. E_1_ and E_2_ are then identified as the resonant energies, Co 2p_3/2_ → unoccupied valence orbitals (L_3_ edge transitions), whereas E_3_ and E_4_ correspond to Co 2p_1/2_ → unoccupied valence orbitals (L_2_ edge transitions). The spectrum at E_1_ in Fig. 2C is seen to also exhibit particularly strong enhancement, by a factor of 30, of the HOMO emission (at 8.5 eV BE, *3a*) as compared to E_2_ and E_3_ excitation; at E_4_ the HOMO signal-intensity is the same as for non-resonant energies. For reference we also show in the bottom tier of Fig. 2C the off-resonant (measured at 778 eV photon energy) PE spectrum of the K_3_[Co(CN)_6_](aq) solution which is largely the spectrum of neat liquid water.

**Figure 3:**
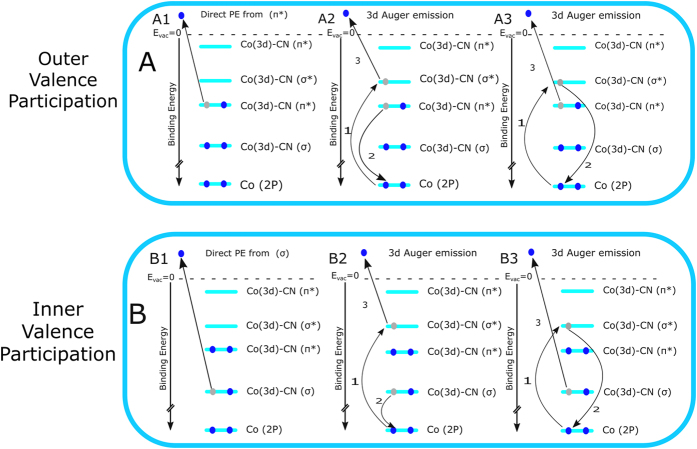
Illustration of the resonantly enhanced photoemission at Co L_3_ edge excitations, (E_1_ and E_2_ in Fig. 2). Outer valence participation (**A**) explains the peak intensities at 8.5 eV ((**C**), feature 3a), and inner-valence participation explains the resonant enhancement at 13.2 eV (Fig. 2C, features ***2a**, **2b***) binding energy. A1 and B1 represent the direct PE from the outer and inner valence orbitals. A2, A3, B2 and B3 depict the Auger contributions to the signal enhancements at E_1_ and E_2_ resonant excitations, respectively. The orbital mixing and bonding nature are also shown. Schematic drawings are created using Inkscape 0.91, https://inkscape.org/en/.

Signal increases in the >30-eV BE range (Fig. 2A,B) result from inelastic electron scattering, reflecting the large number of Auger electrons produced at resonance. As was briefly mentioned signal enhancement at resonant excitation originates from the quantum mechanical interference of electron waves associated with two different photoemission pathways, but leading to the same final state. The situation is illustrated in Fig. 3 for several relaxation processes relevant here. In the top tier (A), Fig. 3-A1 shows the direct ionization of the HOMO of the [Co(CN)_6_]^3−^ complex. At the lowest resonant excitation, Co 2p → LUMO, the processes of Fig. 3-A2, representing participator Auger decay, will result in the emission of Auger electrons with the same kinetic energy as the direct photoelectron. Note that the processes depicted in Fig. 3-A2 differ in the energy levels involved in the relaxation but the net effect, i.e., the energy of the Auger electron, is the same. The analogous situation for deeper-valence ionization/relaxation is presented in the bottom tier (B) of Fig. 3; details are described in the figure caption.

We next assign all peaks that get enhanced upon resonant excitation (Fig. 2A,B) based on the well understood valence-photoionization spectrum of liquid water. An analysis based on ground-state DFT calculations is presented thereafter, in the theory section. For the first point we compare the off-resonant PE spectrum (bottom tier) of Fig. 2C with that of Fig. 1, measured at much lower photon energy. Both spectra are reproduced in Figure SI-1 of the Supporting Information (SI). Differences in the relative intensities of peaks are due to the different cross sections for water ionization at 778 versus 200 eV. Important though, we can accurately reproduce the 778 eV solution spectrum using the same Gaussian peak widths and BE positions of the 200 eV neat water spectrum, if we represent the small extra signal intensities from the solute by four additional Gaussians. Their positions are at 8.5, 10.0, 14.6, and 22.2 eV. The 8.5 eV (HOMO) and the 22.2 eV (K^+^ 3p) peaks have been already identified in Fig. 1. We show below that the 10 eV and 14.6 eV peaks can be attributed to ligand ionization. Signal enhancement at resonant excitation is the reason for observing the 20.5 eV peak (***1a**, **1b***) in the Co 2p RPE spectra (Fig. 2C) but not in the off-resonant spectra (Figure SI-1) in which case the ionization cross section is too small.

### Co L-edge RIXS map and spectra

**Figure 4:**
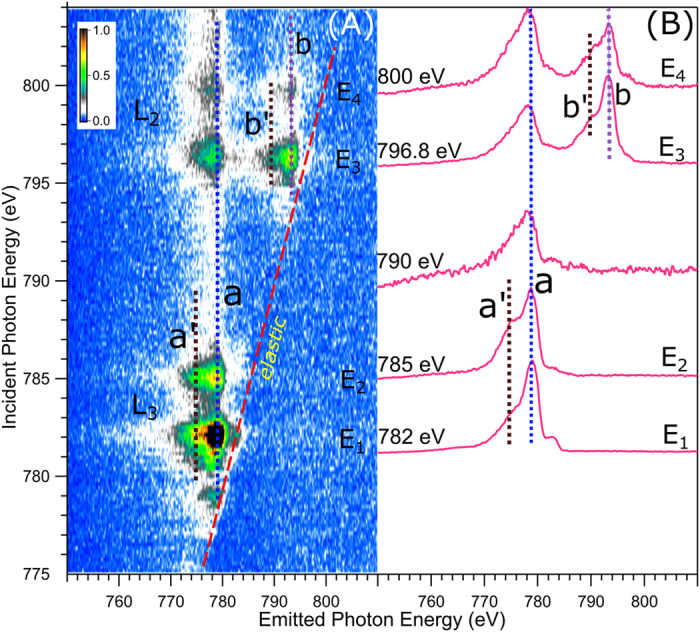
(**A**) Cobalt L edge RIXS plane. The slanted red line shows the elastic spectral line, the vertical dotted blue (***a***) and black (***a*****′**) lines show the constant-energy emission features. (**B**) Selected constant incident energy spectra obtained for a longer duration. E_1_ and E_2_ correspond to the resonant excitations at L_3_ edge, E_3_ and E_4_ corresponds to the L_2_ edge. The spectrum at 790 eV excitation presents the non-resonant XE. All spectra are normalized and shifted along incident photon energy axis. Spectral features at L_2_ edge excitations are shown by dotted lines (***b*** and ***b*****′**).

**Figure 5:**
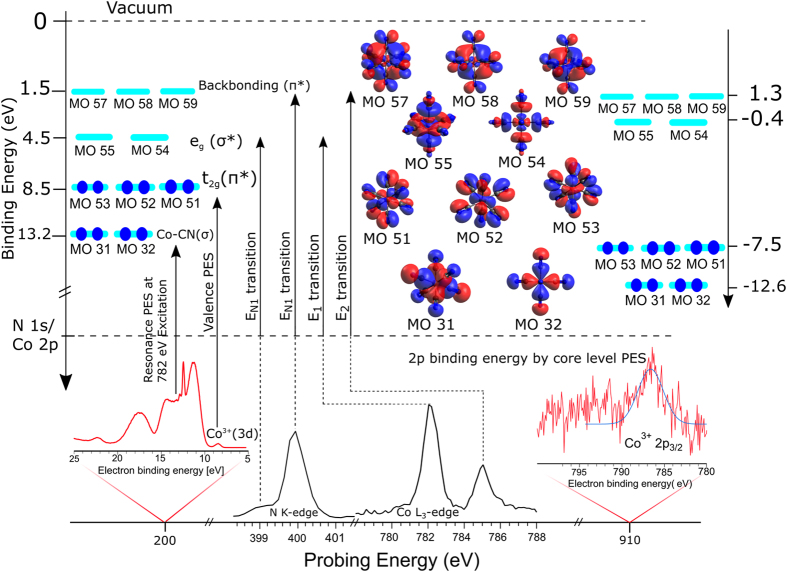
The important experimental and calculated binding energies of K_3_[Co(CN)_6_](aqua) complex. The energy scale on the left side shows the experimentally obtained binding energies inferred from core-level and valence PE spectra, and from PEY-XA spectra. The horizontal axis in the bottom shows the energy ranges used for probing the system. In the middle we illustrate the calculated orbital shapes^46^ of the most important orbitals involved in the processes. On the right side we also present a calculated energy scale (not to scale). Schematic drawings are created using Inkscape 0.91, https://inkscape.org/en/.

Figure 4A presents the Co L_3,2_-edge RIXS plane, *i.e.*, emitted X-ray intensities as a function of excitation and emitted photon energy. Total acquisition time was approximately 175 min, and includes measurement of 150 individual RIXS spectra in the 775–805 eV photon emission range of the 3d–2p transitions. Strongest X-ray emission signal is observed at the same excitation energies E_1_ through E_4_ as for the electron channels (Fig. 2). Corresponding individual RIXS spectra, which are horizontal cuts through Fig. 4A at E_1_, E_2_, E_3_, and E_4_, respectively, are shown in Fig. 4B. Intensities are displayed to yield the same peak height at maximum emission. Positions of the respective elastic peaks, located along the tilted line in the RIXS plane, are used for energy calibration of the emitted-photon-energy axis. The main feature in the L_3_-edge RIXS spectra is an emission peak at 779 eV (label ***a***) and a shoulder at 775 eV (***a***′). Very similar peak shapes are observed at the L_2_-edge (E_3_, E_4_), but in addition a smaller-intensity structure occurs at higher emission energies, peaking at 793 eV (label ***b***) and 789 eV (***b*****′**). Feature ***a*** obviously corresponds to the filling of the Co 2p_3/2_ core-hole by an electron of the occupied valence orbitals, whereas feature ***a*****′**, 4 eV below feature ***a***, must be assigned to an electron transition from some inner-valence orbital of considerable metal character. Features ***b*** and ***b*****′** correspond to the transitions from the outer and inner valence orbitals to Co 2p_1/2_. Note that the energy spacing between ***b*** and ***b*****′** is the same as for ***a*** and ***a*****′**. The latter transitions are indicated in Fig. 5 which is an energy-level diagram of all experimentally inferred cobalt-derived orbitals, but also computed energies as well as orbital characters which are yet to be detailed, are included. The energy axis of Fig. 5 is based on the experimental BEs from Fig. 1 (valence PE spectrum is reproduced at the bottom) and 2, and here we have also included the Co 2p_3/2_ PE spectrum (also presented at the bottom), revealing 786 eV BE. The energy values of ***a*** and ***a*****′** are seen to match the energy differences between the occupied valence and the 2p core level; the same applies for the transitions into Co 2p_1/2_ which are not considered though in Fig. 5. From the value of the Co 2p_3/2_ BE combined with the resonance energies E_1_ and E_2_ (Figs 2 and 4) we find that the binding energies of the relevant unoccupied valence orbitals are 4.5 eV and 1.5 eV. The two excitations are indicated by the vertical arrows in the Fig. 5.

### Co L-edge PEY- and PFY-XA spectra

**Figure 6:**
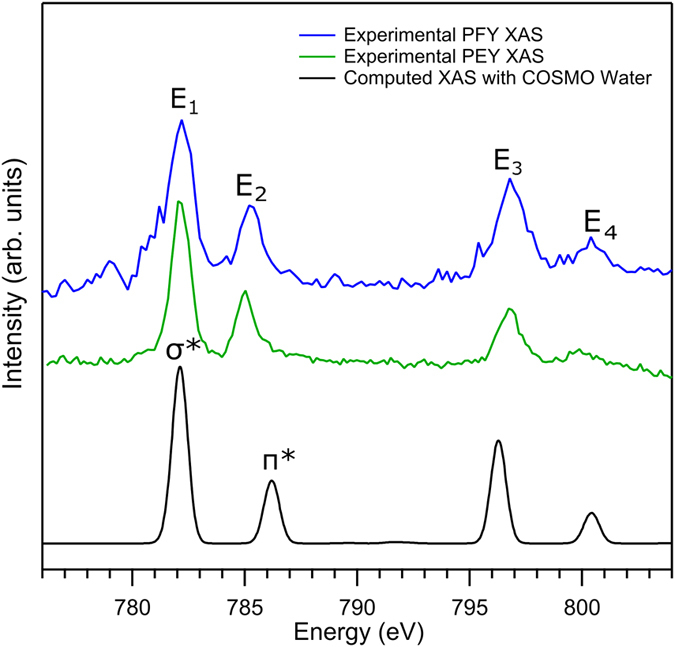
The RIXS and RPE signal integrated Cobalt L-edge (left panel) PFY and PEY XA spectra from 200 mM K_3_[Co(CN)_6_] aqueous solution. The solvent-effect-included calculated XA spectrum is shown in the bottom tier. Important features are marked as E_1_ to E_4_ (see text for more details).

Calculation of RPE and RIXS spectra remains computationally challenging, whereas the resulting PEY/PFY-XA spectra can be simulated with very high accuracy. The experimental XA spectra are obtained by integration of the signal intensities of individual, subsequent RPE/RIXS spectra measured across the resonance. In the present case this corresponds to projecting the total intensity of a given horizontal cut through Figs 2B and 4A, respectively, on the excitation photon energy axis; this however considerably reduces the available spectroscopic information. The resulting partial-yield XA spectra are shown in Fig. 6, PEY-XA in green and PFY-XA in blue. The four prominent absorption bands (E_1_–E_4_) correspond to the E_1_ through E_4_ excitations, respectively, also illustrated in Fig. 5. The computed XA spectrum is shown in black (Fig. 6), and a detailed discussion will be given along with the DFT/ROCIS calculations. The two experimental XA spectra are very similar, and because of the rather high noise level of the PFY-XA spectrum anticipated minor differences arising from detection of non-radiative versus radiative channels^17^ are not resolved. For that reason we attribute the broadening of the E_1_ feature towards lower absorption energies in the PFY-XA spectrum (and absent in the PEY-XA spectrum) to the difference in final states^17^ or to some experimental artifact.

### Nitrogen K-edge RPE map and spectra

**Figure 7:**
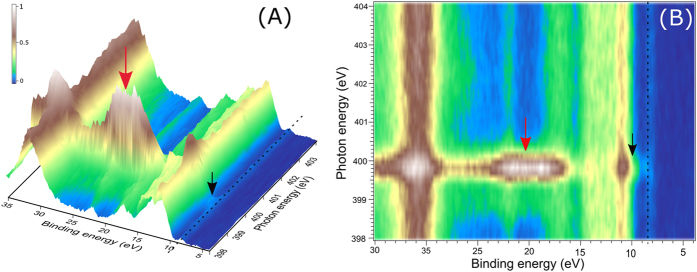
RPE spectra of 200 mM K_3_[Co(CN)_6_] aqueous solution obtained upon tuning the photon energy across the N K-edge. The important features are marked by arrows and dotted lines.

Figure 7 shows the 3-dimensional (in A) and 2-dimensional (in B) resonant valence PE map at the N K-edge from 200 mM K_3_[Co(CN)_6_] aqueous solution; these presentations are fully analogous to Fig. 2A,B for the Co 2p edge. Most noticeable is the electron emission intensity in the 16–24 eV BE range and near 11 eV BE, for 399.8 eV excitation photon energy. The latter coincides with the neat water 1b_1_ BE. Signal around 22 eV BE is convoluted by K^+^ (aq) emission, and signal at 17.5 eV arises from water 1b_2_. There is another signal enhancement near 20.5 eV BE (marked by the red arrow) which is however only revealed when subtracting the non-resonant spectrum from the resonant PE spectrum. Note that enhancement of the 20.5 eV BE peak intensity has been also observed at the Co 2p resonance (Fig. 2C), implying that the respective orbital must be of ligand–metal mixed character. Figure 7 also reveals smaller electron signal, occurring at 8.5 eV BE (marked by black dotted line), and its intensity is also highest for 399.8 eV excitation energy. This peak arises from HOMO ionization of the metal complex as we have already discussed (see Fig. 5). At the same excitation energy we also observe a slightly enhanced electron emission near 11 eV BE (marked by the black arrow) which appears to coincide with the 10 eV peak identified in the off-resonant valence PE spectrum of Figure SI-1. Since this peak is not enhanced in the Co 2p RPE spectra of Fig. 2C, this feature must be of nitrogen character. Qualitatively, in accordance with our previous valence PE spectroscopy studies on [Fe(CN)_6_]^4−^(aq)^39^, the 10 eV peak can be assigned to the CN^−^ π-orbitals. We will show that this is also in agreement with our calculations.

### Nitrogen K-edge RIXS map and spectra

**Figure 8:**
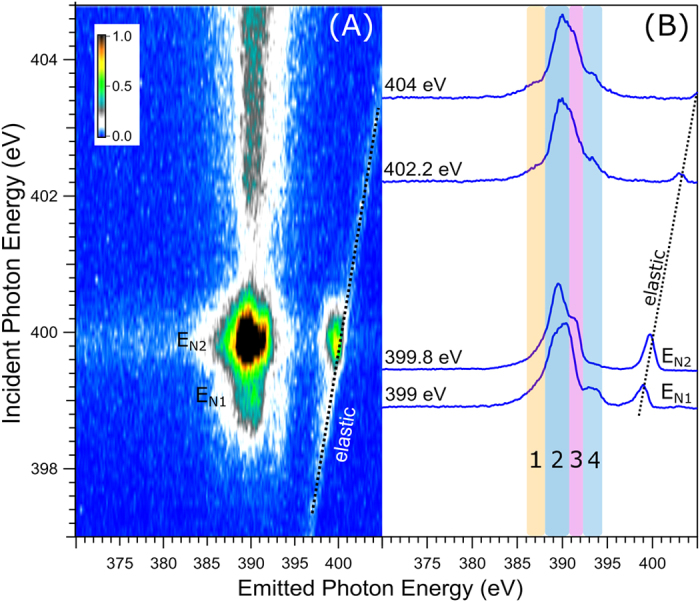
(**A**) Nitrogen K-edge RIXS plane. The slanted black dotted line corresponds to the elastic emission. (**B**) 1D spectra excitation energies E_N1_ and E_N2_; acquisition time was 20 times longer than for the respective traces of the RIXS plane.

Figure 8A presents the N K-edge RIXS plane including 100 individual RIXS spectra; total acquisition time was 140 min. As for the Co 2p RIXS map (Fig. 4A), the signal along the slanted line corresponds to the elastic peak, and serves for energy calibration. Strongest emission intensities are observed in the approximately 385–395 eV range, at the resonant excitations 399.0 eV (denoted E_N1_) and 399.8 eV (E_N2_). Weaker emission intensity in the same energy range is observed for excitation photon energies larger than 402 eV. Increase of the elastic peak intensity at the E_N2_ resonance originates from a larger probability for filling the 1 s core-hole by direct decay of the excited electron^40^, which is accompanied by the enhancement of Auger decay, as seen in Fig. 7. As for the Co 2p RIXS data, we focus our discussion of Fig. 8A on the most noticeable emission features by considering just a few selected RIXS spectra, measured at E_N1_, E_N2_, and at two energies, 402.2 and 404.4 eV; the latter correspond to electron excitation into the continuum. The spectra are shown in Fig. 8B. Signal intensities are displayed to yield the same peak height at maximum emission. All spectra are seen to exhibit a similar 385–395 eV broad-band emission, with the main difference being the distribution of relative intensities within the band. We can use the latter to divide the band into four spectral regions, ***1***-***4***, as indicated in Fig. 8B. Lowest-energy feature ***1*** has small intensity; it yet becomes stronger and more pronounced when going from 399/399.8 eV to 402.2/404 eV resonant excitation. Feature ***2*** has largest intensity in all spectra, and spectral shape stays largely unchanged at all excitation energies. Feature ***3*** is a shoulder (overlapping with ***2***) of varying intensity, and only at 399.8 eV (E_N2_) it forms a rather distinct peak; intensity is lowest at 399 eV (E_N1_). Feature ***4*** is an only slightly larger peak than 1. It nearly vanishes at E_N2_. Our spectral division into ***1***-***4*** is indeed meaningful and correlates with our non-resonant XE-spectra calculations shown in Figure SI-4, and also further detailed in the theory section below.

### Nitrogen K-edge PEY- and PFY-XA spectra

**Figure 9:**
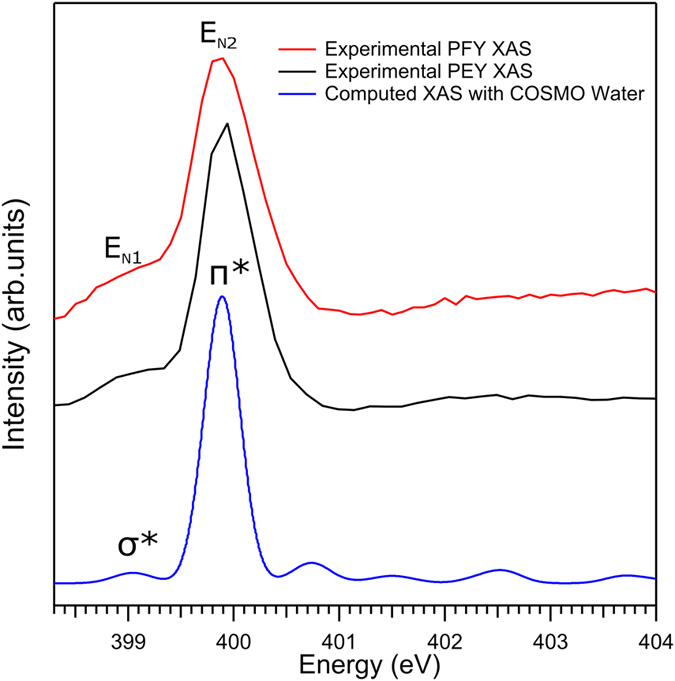
The RIXS and RPE signal integrated N K-edge PFY- and PEY-XA spectra (red and black tiers, respectively). The blue spectrum at the bottom is obtained from TDDFT calculation. E_N1_ and E_N2_ mark the resonance energies; same as in Fig. 8.

The experimental PFY- and PEY-XA spectra for the N K-edge are shown in Fig. 9 along with a calculated spectrum (bottom tier) where solvent effects have been included. XA spectra are generated from Figs 7 and 8, analogous to the procedure explained for the Co 2p PY-XA spectra. The two experimental spectra exhibit the very similar broad single absorption band at 399.8 eV (E_N2_) and a wide shoulder near 399.0 eV (E_N1_); possible spectral differences arising from photon versus electron detection^41^ cannot be quantified here as improved signal-to-noise level (particularly of the data shown in Fig. 8A) would be needed. Qualitatively, the absorption at E_N1_ and E_N2_ corresponds to the promotion of an N 1 s electron to unoccupied valence orbitals as indicated in Fig. 5.

### Results from electronic-structure calculations

#### Energy and character of metal-derived occupied valence orbitals

For a theoretical interpretation of the peaks at 8.5 eV (***3a***) and 13.2 eV (peaks ***2a**, **2b,***) BE in the Co 2p RPE spectra of Fig. 2C (see also Fig. 5) we have performed ground-state single-point DFT calculations to reveal the nature of the corresponding occupied molecular orbitals. Computed orbital energies as well as the relative atomic characters, i.e., percentages of Co, N, and C from Löwdin population analysis, are presented in Figure SI-2A. We have also analyzed the particular nature of the metal 3d orbitals, and relative contributions from d_xy_, d_xz_, d_yz_, d_z2_, and d_x2-y2_ are depicted in Figure SI-2B. The calculated orbital energies were shifted by +1.08 eV to match the experimental BE values. Figure SI-2B reveals the expected dominant Co 3d character of the HOMO originating from the hybridized Co–C π* orbitals. This is inferred from the fact that we find only contributions from d_xy_, d_xz_, and d_yz_ which are the antibonding (π*) t_2g_ symmetry orbitals. These are the molecular orbitals (MO) #51, 52, 53 in our calculations; the respective orbital shapes are shown at the right side in Fig. 5. The photoemission occurring at 13.2 eV in the experiment is argued to correspond to the orbitals calculated at 12.6 eV. As seen in Figure SI-2A these molecular orbitals, #31, 32, have mainly cyanide character (≈65%) and smaller Co 3d character, with the latter being comprised of d_z2_ and d_x2-y2_ orbitals, these are the hybridized Co–CN σ-type bonding orbitals.

#### Co L-edge: Interpretation of RIXS spectra assisted by XE-spectra calculation

Non-resonant X-ray emission (XE)-spectra calculations have been performed based on transition-dipole-moment calculations between the MOs. The calculated spectrum (Figure SI-3) is found to reasonably well reproduce the experimental 790 eV spectrum of Fig. 4B. Four sets of transitions centered at 774.2, 776.0, 776.2, and 778.2 eV are found to contribute with different weights. Energy positions are given by the sticks in Figure SI-3, and probabilities are indicated by the heights of the sticks. Representing each stick by a Gaussian with 1 eV width then yields the computed XE spectrum in Figure SI-3. Our calculations reveal that the strongest emission in the experimental spectra, at 779 eV (transition ***a*** in Fig. 4B), results from HOMO-2, HOMO-1, and HOMO (MOs #51-53) to Co 2p_3/2_. The respective orbitals and their energies are indicated in Fig. 5. The transitions at the calculated energies 776.0/776.2 eV can be connected with the two sets of Co–C π (#33, #34, #35) and Co–C σ* (#37, #38) orbitals. The orbitals responsible for transition ***a*****′** (calculated at 774.2 eV) can be traced to orbitals #31, 32 which we had already identified as Co-CN σ-type bonding orbitals. According to Fig. 5 the experimental energy difference between ***a*** and ***a*****′** is approximately 4.7 eV which is close to the 5.1 eV value obtained in the ground-state energy calculation. The calculated energy difference based on the excited-state XES calculations is only 4 eV (not shown in Fig. 5) which can be attributed to an overestimation of correlation between valence-to-core electrons in the presence of a core hole.

**Figure 10:**
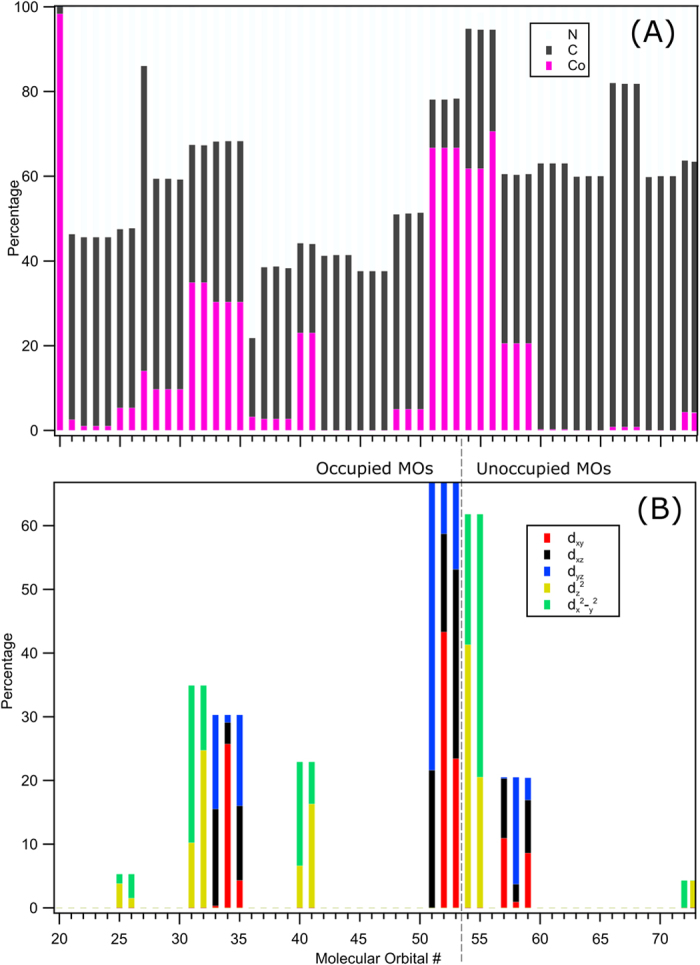
Löwdin population analysis for [Co(CN)_6_]^3−^ obtained from single point DFT calculations. The top tier (**A**) presents different atomic contributions, and the bottom tier (**B**) shows the Co 3d contribution to the various MOs. The unoccupied and occupied MOs are separated by the black dotted line between MOs #53 and #54.

We next explore the actual character of the orbitals relevant for the interpretation of the main features of the experimental XE spectra (Fig. 4B) in more detail. In Fig. 10A we present the relative element-specific contributions to a given MO, and Fig. 10B details the contributing Co 3d character in terms of d_xy_, d_xz_, d_yz_, d_z2_, and d_x2-y2_ orbitals, both obtained from Löwdin population analysis^42^. The small shoulder at 775 eV emission in the experimental XE spectrum (feature ***a*****′** in Fig. 4B) can be associated with orbitals which are of Co–CN σ bonding character, according to Fig. 10A, this involves MOs #31 and #32 with approximately 35% Co (3d-e_g_) character. The two close-lying calculated transitions at 776 and 776.2 eV (Figure SI-3) originate from MOs #33 to #39, all of which have dominant CN^−^ ligand character, approximately 70% in MOs #33–35 and 95% in MOs #36–39. Analogously, HOMO, HOMO-1 and HOMO-2 which we attribute to the strongest feature at 779 eV, ***a***, are of Co-CN π* anti-bonding character. These MOs are #53, #52 and #51 and have approximately 65% Co (3d-t_2g_) character, consistent with their intense contribution in the Co RIXS spectra.

#### Nitrogen K-edge: Interpretation of RIXS spectra assisted by XE-spectra calculations

The calculated N XE spectrum along with the experimental spectrum measured at 404 eV excitation energy, is shown in Figure SI-4M. The calculations reveal four groups of transitions, near 387.8, 390, 391, and 392 eV, very well matching our division into spectral regions ***1***-***4*** which was introduced in Fig. 8. In Figure SI-4 computed energy positions are presented by sticks, and the respective transition probabilities are given by the stick heights. Representing the individual sticks by Gaussians, and applying 1 eV broadening, the theoretical (green) spectrum is obtained which is in fairly good agreement with the experimental 404 eV XE spectrum of Fig. 8B. Based on our calculations X-ray emission feature ***1*** is attributed to the refill of the nitrogen 1 s core-hole by electrons from the mixed Co (3d-e_g_) and CN^−^ orbitals which have σ-character (#31, #32). The low intensity of ***1*** is attributed to the considerable Co (3d-e_g_) contributions to these valence orbitals which is supported by Fig. 10 (see orbitals #31 and #32). The main emission-feature ***2*** is attributed to the relaxation of electrons (nearly 100%) from CN^−^ orbitals to the N 1 s core-hole. The orbitals responsible for emission in this energy are MOs #36–39 as can be seen from Fig. 10. For feature ***3*** we observe three transitions (see the blue sticks near 391 eV in Figure SI-4). The orbitals emitting in this range have mainly CN^−^ character, and Co 3d contributions are very minor (Fig. 10, orbitals #42–50). The reason for the intensity enhancement of ***3*** at E_N2_ resonant excitation (Fig. 8B) is not understood. Finally, emission ***4*** which is strongly enhanced at E_N1_ excitation is found to originate from the Co–CN outer-valence orbitals, HOMO-2, HOMO-1, and HOMO (MOs #51-53) which have more than 65% Co (3d-t_2g_) contributions (see Fig. 10A).

#### Computed Co L-edge PEY and PFY-XA spectra

With the aid of our DFT/ROCIS calculations, which well reproduce the experimental XA spectra (see black curve in Fig. 6) we can now accurately assign absorption features to their respective transitions. The first intense peak E_1_ (782 eV) is thus attributed to the 2p → σ*(e_g_) transitions; these MOs are the two lowest unoccupied molecular orbitals (LUMO, LUMO + 1) of the molecule. This transition is indicated in the energy-level diagram of Fig. 5. Here the energy of the 786.5 eV BE of Co 2p_3/2_ has been taken from the respective core-level PE spectrum shown in Figure SI-7, and the spectrum is also reproduced at the bottom of Fig. 5. The second peak E_2_ (785 eV) in Fig. 6 can be assigned to 2p → π* transitions, which corresponds to π-backbonding, characteristic of transition-metal complexes with strong field ligands. The origin of this feature is the mixing of Co 3d t_2g_ orbitals with the empty 2π* orbitals of CN^−^ forming an occupied deeper valence bonding orbital and an unoccupied anti-bonding valence orbital of the same symmetry. The computed orbital shapes of the π* orbitals (#57–59) as well as the respective experimental and theoretical binding energies are also shown in Fig. 5. We note that the σ* and π* band positions (peaks E_1_ and E_2_; see also the assignments made in Figure SI-5) are in reasonable agreement with aforementioned total-electron yield (TEY) XA spectra from powdered samples^14^. However, the L_2_ peak in the present work is shifted to 1.2 eV lower energy compared to the powder study^14^, which may be indicative of a solvent effect.

#### Qualitative probing of bonding strength – N K-edge PEY and PFY-XA spectra and theory

The strength of the π-backbonding is determined by the extent of orbital mixing, and this should be reflected in the intensity of the respective absorption bands. Probing π-backbonding will hence depend on the particular excitation. For instance, if the (unoccupied) orbitals responsible for the π-backbonding have dominant metal character we expect that the respective spectral contributions are larger for the metal → π* than for ligand → π* transitions. This provides a spectroscopic means for the qualitative analysis of the strength of bonding and will be discussed next.

In order to evaluate the effect of metal versus ligand excitation on the σ*-to-π* band intensity ratio we must assign the experimental nitrogen K-edge PFY-XA and PEY-XA spectra of Fig. 9 with the help of our TDDFT calculations. The computed XA spectrum, with solvent effect included, which has already been included in Fig. 9, is seen to be in good agreement with the two experimental spectra. Based on the calculations the first absorption E_N1_ can be assigned to the N 1s → σ*(e_g_) transition, involving population of MOs #54 and #55. The main peak E_N2_ corresponds to the N 1 s → π* transitions, and involves population of MOs #57–59; both N 1 s transitions are indicated in the energy-level diagram of Fig. 5. Comparing the nitrogen K-edge XA spectra (Fig. 9) and the Co L-edge XA spectra (Fig. 6), we find that the σ*-to-π* band intensity ratio is considerably different for the two cases. The larger ratio in Fig. 6 immediately suggests that the σ* orbitals have dominant metal character, whereas the π* orbitals have dominant ligand character indicated by the intensity ratio in Fig. 9. This is indeed confirmed by our orbital analysis presented in Fig. 10A. As can be seen, MOs #54 and #55 have 60% Co contribution but MOs #57-59 have only 20% metal character.

An important aspect related to the comparison between experimental and computed XA spectra is whether there is any evidence for (water) solvent-specific effects. One experimental indication might be the aforementioned shift in the L_2_ peak position when comparing XA spectra from aqueous solution with powder samples. Another observation is that the experimental XA spectra, both for the Co L-edge and the N K-edge, are best reproduced by calculations with the solvent effect included. As shown in Figure SI-5 for the Co XA spectra, the π* band is shifted to considerably lower excitation energy, in better agreement with experiment, when the solvent is taken into account. This is similarly true for the nitrogen spectra, where also better agreement between experiment and computation is obtained when the solvent effect is included; results are presented in Figure SI-6.

**Figure 11:**
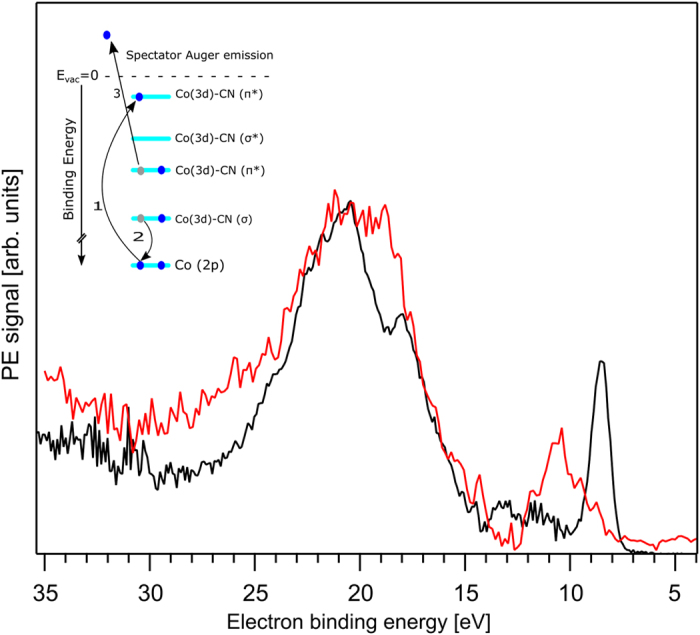
Resonant photoelectron spectra of aqueous [Co(CN)_6_]^3−^ at 785 eV (Co L-edge) and 399.8 eV (N K-edge) photon energy after subtraction of an off-resonant PE spectrum measured several eV below the respective absorption edge (see text for details). The intensity of the Co RPE spectrum has been increased by a factor of 10 to match with the intensity of the N 1s RPE spectrum. The inset in the figure shows the Auger-emission channel responsible for the signal enhancement around 20.5 eV BE. Schematic drawings are created using Inkscape 0.91, https://inkscape.org/en/.

We have so far explored how the metal versus ligand (local) excitation reflects in the respective XA spectra. Experimentally, this information can be inferred in a straight-forward manner, as we have shown, and on the theory side XA spectra calculations can be performed rather accurately. More detailed information is however contained in the RPE spectra which reveal the actual energies of overlapping ligand and metal orbitals through measured signal enhancements due to interfering electron emission channels. This has been recently demonstrated for Fe^3+/2+^ and Ti^3+^ aqueous solutions^41^,^43^,^44^. For the present study, where we are interested in different local-excitation probes, we thus compare the valence RPE spectra measured at the Co L-edge (already shown in Fig. 2C) and at the nitrogen K-edge. Both spectra are presented in Fig. 11, and from each an off-resonant (at few eV below the respective resonance; 785 eV for Co (E_2_) and 399. 8 eV for N (E_N2_)) PE spectrum has been subtracted in order to single out the signal enhancements at resonance. Both difference spectra are displayed to have the same peak heights at maximum signal, near 20 eV BE. Note that the absolute signal intensity for the N-edge-resonance spectrum is approximately ten times higher which is due to the larger cross section for N 1s absorption, and the six times larger number of nitrogen atoms compared to Co. Both spectra exhibit a rather similar broad electron emission in the approximately 13–25 eV BE range, with one noticeable difference being an intensity dip near 19 eV BE in the Co spectrum. Spectral differences are very pronounced however in the <15 eV BE region, where large relative intensity variations of the various peaks discussed in the previous sections are observed. Specifically, we detect larger (relative) intensities for peaks 8.5 eV (#51–53) and 13.2 eV (#31, #32) BE for the Co resonance, and indeed these emissions correspond to orbitals with mainly metal character (compare Fig. 2C). On the other hand we find larger signal near 10 eV BE for the N 1s excitation, and this is consistent with our above assignment that the respective orbitals (#42–44) have mainly ligand character. The large 15–25 eV electron emission band can be assigned to spectator Auger processes which involve 2-hole 1-particle final states. This can explain why we cannot assign specific orbitals derived from ground-state calculations for the transitions in this energy range, as spectator Auger transitions constitute a large departure from the electronic ground state. The situation is depicted for the deeper valence Co-CN(σ) to Co 2p spectator Auger refill in the inset of Fig. 11. On the other hand, large signal intensity of the HOMO at 8.5 eV BE is attributed to participator Auger decay, in which case the kinetic energy of the Auger electron is the same as for the photoelectron at this resonant photon energy. An analogous explanation holds for the 10 eV peak, for the N 1s participator Auger decay. Note that the latter process is not depicted because the N 1s BE of [Co(CN)_6_]^3−^(aq) has not been detected in this study. The differences in spectral shape at energies above 25 eV in Fig. 11 are attributed mainly to the different kinetic-energy dependent cross sections for the inelastically scattered photoelectrons in the solution.

## Conclusions

RPE and RIXS spectra unambiguously reveal orbital mixing between metal center and CN ligands in the aqueous [Co(CN)_6_]^3−^ complex. Strong π-backbonding, i.e., back-donation of the metal t_2g_ electrons into the empty π* orbitals of the CN^−^ is observed from the σ*-to-π* band signal intensity ratios in the respective partial-yield XA spectra which vary dramatically for nitrogen versus cobalt core-level excitation. The assignment of these bands foots on electronic structure calculations based on DFT/ROCIS and TDDFT. In the case of Fe(CO)_5_ the charge-transfer peak is dominating in intensity compared to the e_g_ peak. This is due to the strong π-acceptor nature of the CO ligand. Also note that the CO ligand is charge-free whereas the cyanide carries a negative charge, and this could partially hinder the charge transfer to the ligand sites^45^. We conclude this section by noting that the strength of back bonding decreases in the following order of metal complexes: Fe(CO)_5_ > [Fe(CN)_6_]^4−^ > [Co(CN)_6_]^3−^. However, a major finding of the present study is that orbital mixing, and even the dominant orbital character, as well as the absolute energy of these orbitals can be accessed from the RPE spectra. This is due to the signal enhancements of peaks in the valence spectra, and these effects depend sensitively on whether the excitation is localized rather on the metal or on the ligand. Our study not only shows that partial electron and fluorescence yield XA spectra reveal important differences due to the particular electron relaxations but it also shows the need to invoke the respective RPE and RIXS spectra to interpret the derived XA spectra. We emphasize the importance of measuring and analyzing RPE and RIXS spectra simultaneously, a significant improvement over previous works employing only electron^39^,^43^ or only photon^9^,^10^ detection methods, and we expect that these complementary techniques will be routinely applied in future electronic-structure investigations of relevant aqueous molecular systems.

## Additional Information

**How to cite this article**: Lalithambika, S. S. N. *et al*. Chemical bonding in aqueous hexacyano cobaltate from photon- and electron-detection perspectives. *Sci. Rep.* **7**, 40811; doi: 10.1038/srep40811 (2017).

**Publisher's note:** Springer Nature remains neutral with regard to jurisdictional claims in published maps and institutional affiliations.

## Supplementary Material

Supporting Information
