# Supplementary material for: Chemical bonding in aqueous hexacyano cobaltate from photon- and electron-detection perspectives
Source: Sci Rep. 2017 Jan 18;7:40811. doi: 10.1038/srep40811 (PMC5241694; doi:10.1038/srep40811)
Supplement: Supporting Information [file srep40811-s1.pdf]

Supporting Information for "Chemical bonding in aqueous hexacyano cobaltate from photon- and electron-detection perspectives"

Sreeju Sreekantan Nair Lalithambika,[a,b] Kaan Atak,*[,a,b] Robert Seidel,[a] Antje Neubauer,[a] Tim Brandenburg,[a,b] Jie Xiao,[a] Bernd Winter,[a] and Emad F. Aziz[a,b,c]

[a] *Institute of Methods for Material Development, Helmholtz-Zentrum Berlin für Materialien und Energie, Albert-Einstein-Strasse 15, 12489 Berlin, Germany*

[b] *Freie Universität Berlin, Fachbereich Physik, Arnimallee 14, D-14195 Berlin, Germany*

[c] *School of Chemistry, Monash University, Clayton 3800, VIC, Australia*

*[*]**Corresponding Author**: kaan.atak@helmholtz-berlin.de*

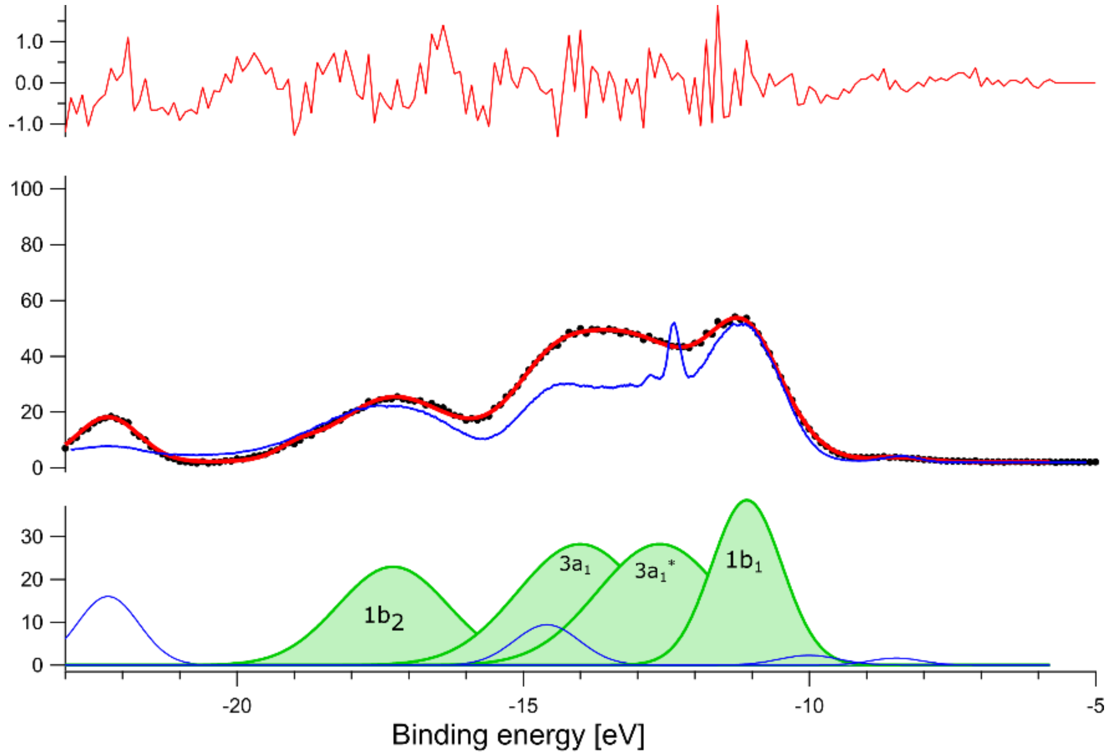

$3a_1$

Figure SI 1. Valence off-resonant PE spectra from 200 mM $K_3[Co(CN)_6]$ aqueous solution measured at 778 eV photon energy (black dotted line) and valence PE of neat liquid water measured at 200 eV photon energy (blue). The green peaks are Gaussians representing the energy positions and width of the PE peaks due to ionization of the water orbitals, $1b_1$, $3a_1$, and $1b_2$. Here, intensities of these Gaussians were allowed to vary in order to fit the 778 eV solution spectrum. A perfect fit is however only possible if four additional Gaussians, representing solute signal contributions are introduced. These peaks are shown in blue. Their positions are at 8.5, 10.0, 14.6, and 22.2 eV BE. The 8.5 eV peak is due to ionization of the HOMO of $[Co(CN)_6]^{3-}$ (aq), and 22.2 eV arises from ionization of K^+ 3p (aq). The peak at 14.6 eV must be attributed to some deeper-lying state of $[Co(CN)_6]^{3-}$(aq).

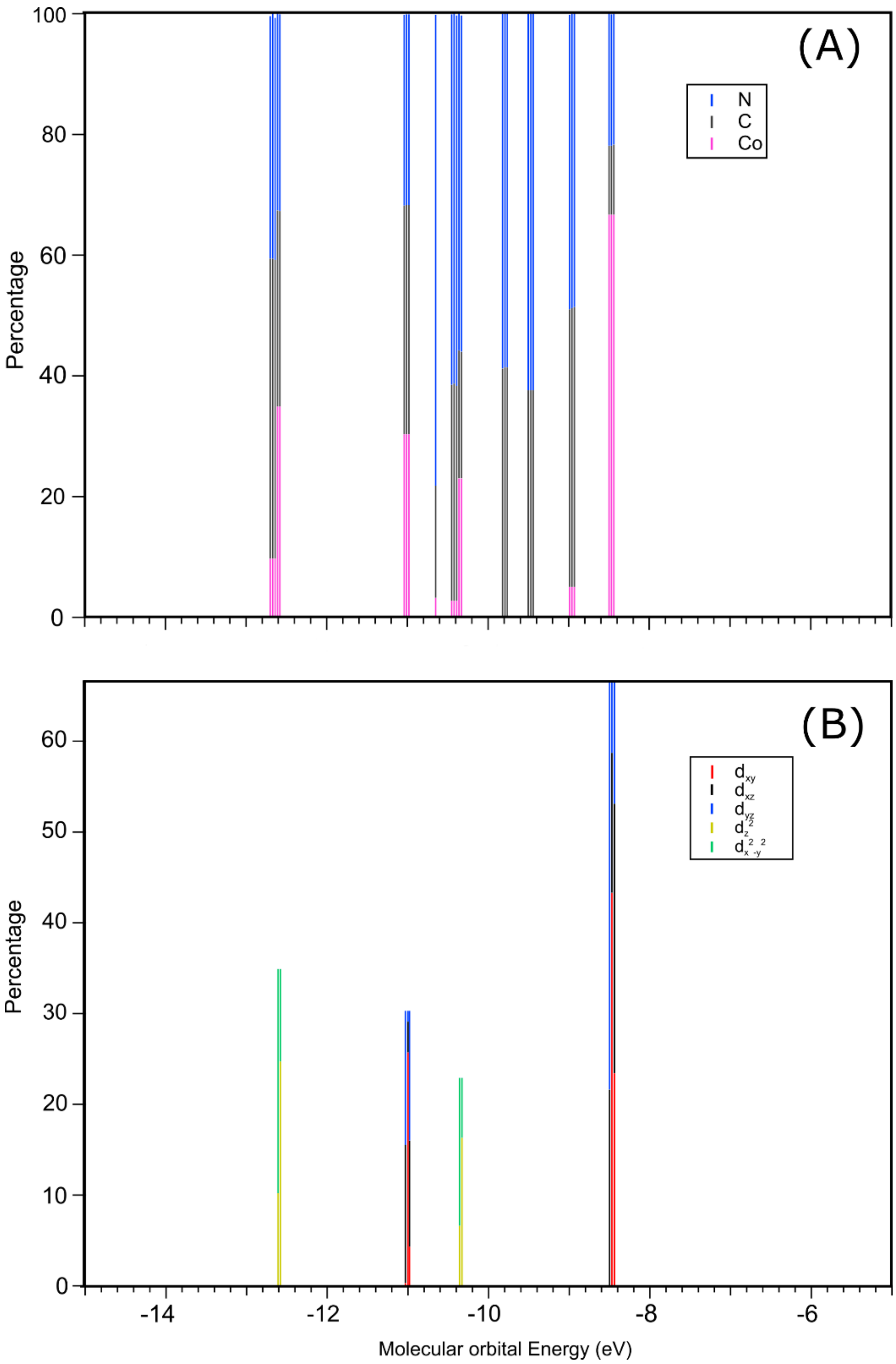

Figure SI 2. Computed valence Löwdin population analysis in molecular orbital energy scale. (A) Presents the contributions from Co (Pink), C (grey) and N (blue). (B) Gives the molecular orbital participation from Co d orbitals alone. An energy offset of 0.03 eV is introduced to make the degeneracies more comprehensible.

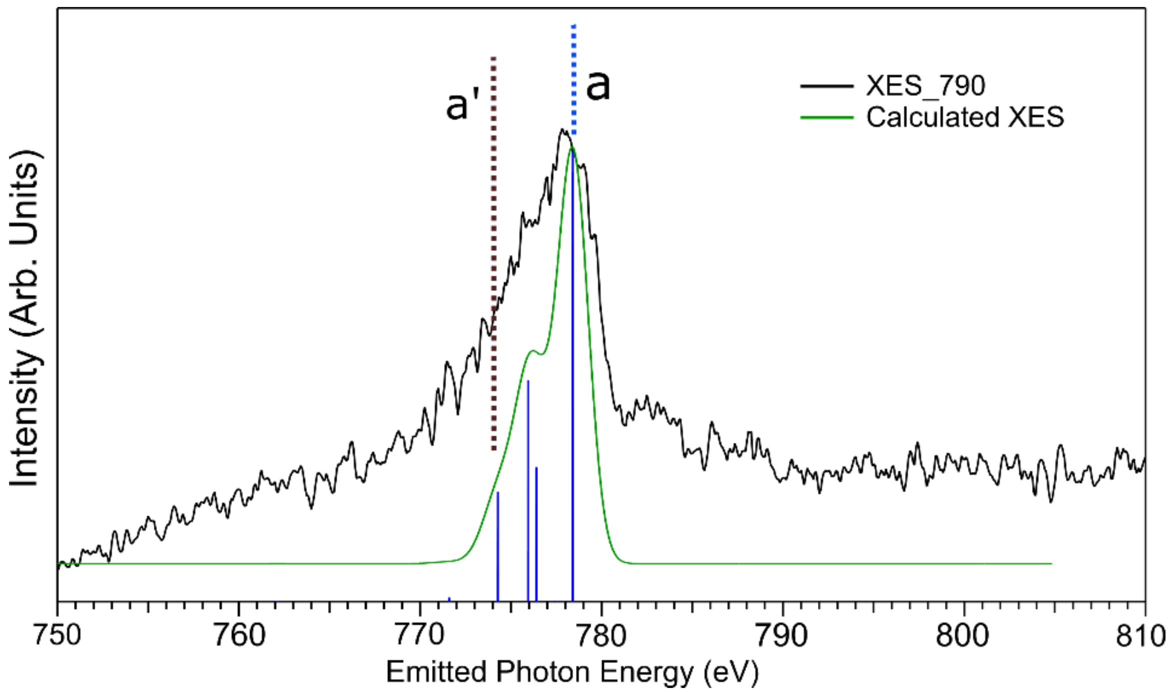

Figure SI 3.

The off resonant experimental cobalt XE spectra from 200 mM $K_3[Co(CN)_6]$ aqueous solution at 790 eV compared with the calculated spectrum. A Gaussian broadening of 2 eV was used for the calculated spectrum.

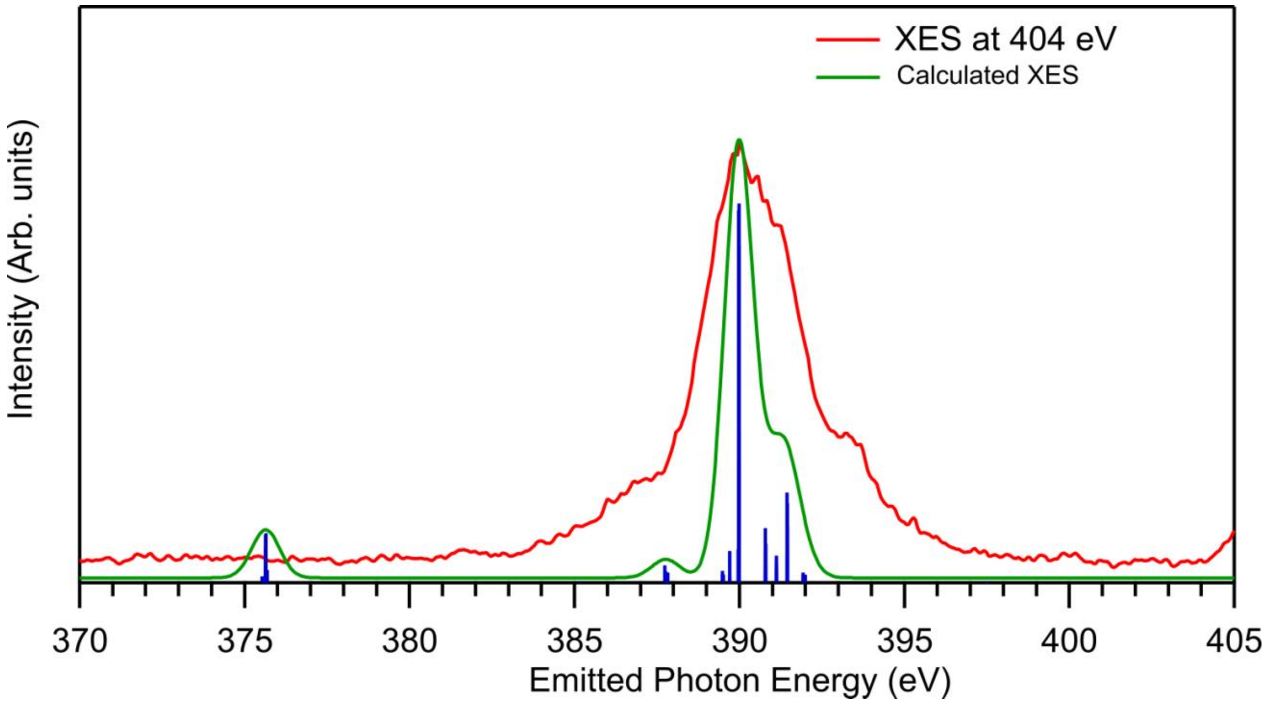

Figure SI 4. Comparison between the experimental XES (red) from 200 mM $K_3[Co(CN)_6]$ aqueous solution at 404 eV excitation with the calculated XES (green). A Gaussian broadening of 1 eV was used for the calculated spectrum and has been shifted by 12 eV to match the experimental spectrum. The calculated spectrum shows a feature around 375.8 eV, but experimental spectrum does not show this feature.

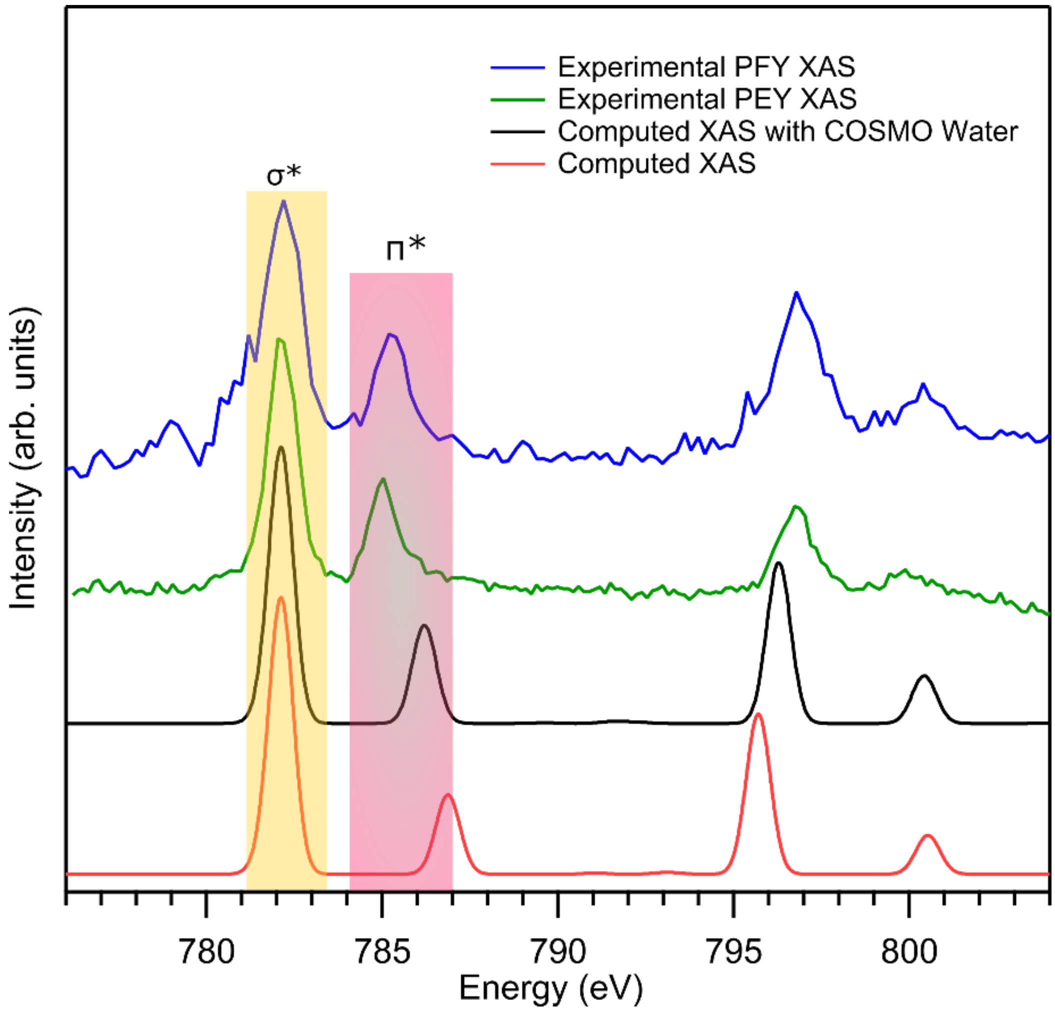

Figure SI 5. Experimental cobalt L edge XA spectrum from PFY and PEY measurements from 200 mM $K_3[Co(CN)_6]$ aqueous solution, along with computed XA spectrum with spin orbit coupling included DFT/ROCIS calculations. The spectrum on the lower tier (red) shows the calculated spectrum without the solvent effect, the notable difference is the position of the transition to the π* molecular orbitals.

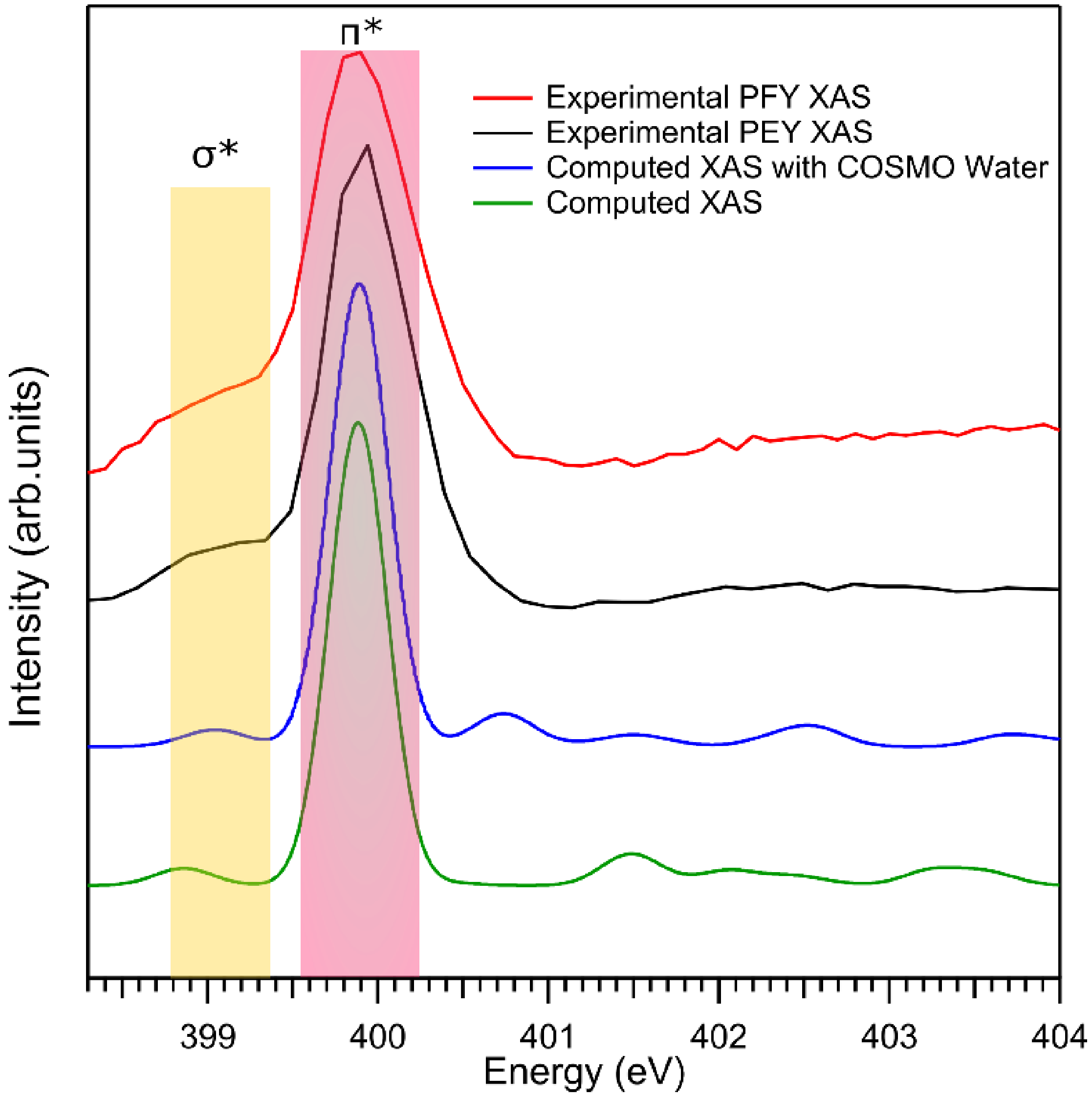

Figure SI 6. Experimental N K-edge XA spectra from PFY and PEY measurements from 200 mM $K_3[Co(CN)_6]$ aqueous solution, along with computed XA spectra using TDDFT calculations. The blue and green spectra illustrate the computed XA with and without solvent effect respectively. The solvent effects were included using the COSMO water function in the ORCA program package.

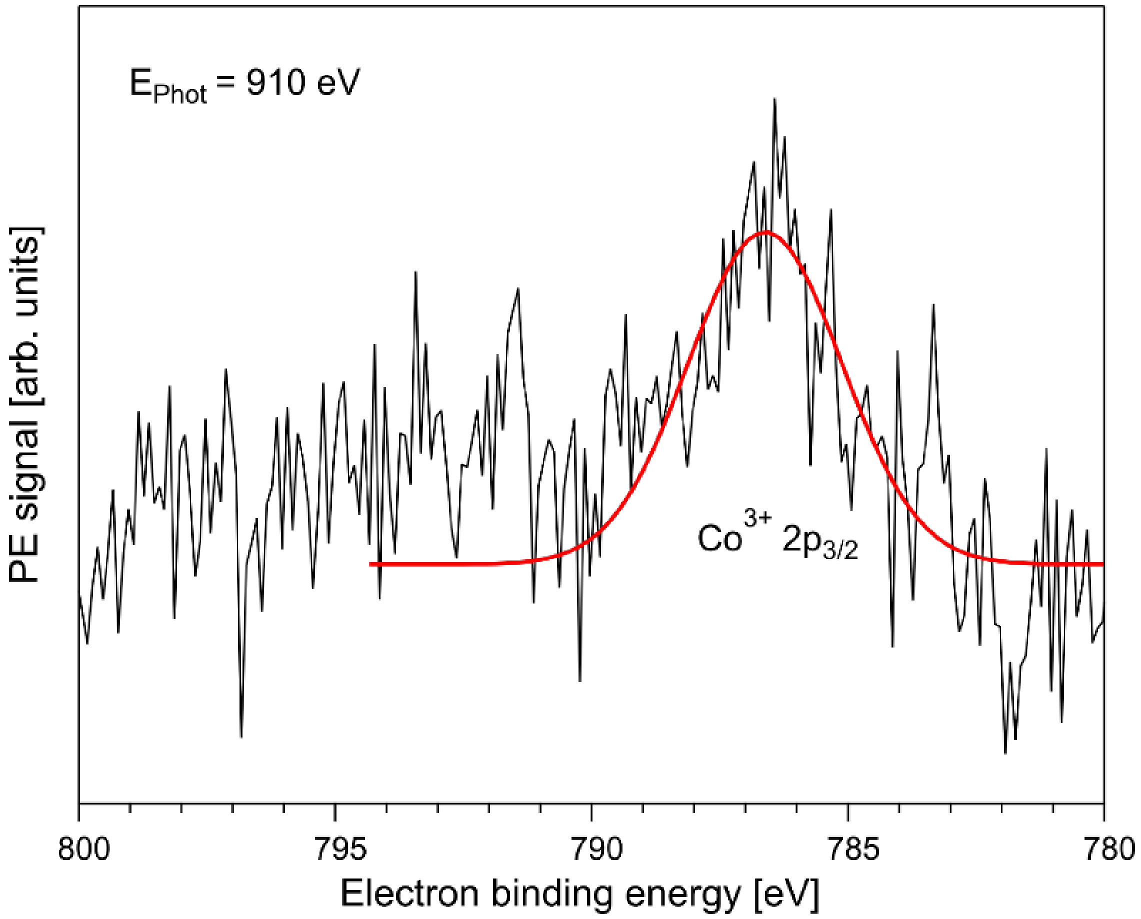

Figure SI 7. Co $2p_{3/2}$ binding energy of 200 mM aqueous $[Co(CN)_6]^{3-}$ solution obtained from core PE spectra measured at 910 eV photon energy.
